# Hippo and Wnt as Early Initiators: Integrated Multi-Omics Reveals the Signaling Basis for Corona-Induced Diapause Termination in Silkworm

**DOI:** 10.3390/insects17010123

**Published:** 2026-01-21

**Authors:** Quan Sun, Xinghui Liu, Guizheng Zhang, Xinxiang Chen, Wenxin Xie, Pingyang Wang, Xia Wang, Qiuying Cui, Yuli Zhang

**Affiliations:** 1 Chongqing Key Laboratory of Big Data for Bio Intelligence, School of Life Health Information Science and Engineering, Chongqing University of Posts and Telecommunications, Nan’an, Chongqing 400065, China; liuxinghui63@gmail.com (X.L.); ephemeralhlh@gmail.com (X.C.); xiewenxin0622@163.com (W.X.); 2 Guangxi Key Laboratory of Sericultural Genetic Improvement and Efficient Breeding, Nanning 530007, China; zhangdoudou1999@163.com (G.Z.); wangpingyang.yczg@163.com (P.W.); xiaw12@163.com (X.W.); 13036887765@163.com (Q.C.)

**Keywords:** corona treatment, diapause, transcriptomic, proteomic, silkworm

## Abstract

Embryonic diapause, a state of developmental arrest in silkworm (*Bombyx mori*) eggs, poses a challenge for year-round sericulture. While physical stimuli like corona discharge can effectively terminate diapause, the underlying molecular mechanisms, particularly the initial events, remain poorly understood. This study employed an integrated transcriptomic and proteomic approach to analyze silkworm eggs within 48 h after corona treatment. Our time-series analysis revealed that the Hippo and Wnt signaling pathways were specifically activated as early as 1 h post-treatment, preceding the previously reported FoxO pathway response. We identified two temporally distinct gene clusters within the Hippo pathway, including immediate–early genes (e.g., *Dachs_17/25/29*, *Ft_10*) and late-phase effector genes, coordinating the exit from cell cycle arrest. Concurrently, the Wnt pathway was rapidly initiated, marked by the sustained upregulation of key regulators *Notum* and *Pontin52*, suggesting its role in unlocking the cell cycle. We propose a synergistic model wherein corona discharge triggers the concurrent, early activation of Hippo and Wnt signaling, which collectively reprogram the cell cycle and reinstate the developmental trajectory by promoting proliferation and suppressing apoptosis. These findings provide crucial insights into the initial molecular events of diapause termination, identifying Hippo and Wnt pathways as master regulators in transducing the physical corona stimulus into a developmental signal.

## Simple Summary

Embryonic diapause in silkworm eggs is a state of developmental arrest that challenges year-round silk production. Although physical treatments such as corona discharge can break diapause, the early molecular events remain unclear. In this study, we used transcriptomic and proteomic approaches to analyze silkworm eggs within 48 h after corona treatment. We found that the Hippo and Wnt signaling pathways are activated as early as 1 h post-treatment, earlier than the previously reported FoxO pathway. These pathways appear to coordinate cell cycle re-entry and developmental resumption. Our findings suggest that Hippo and Wnt act as initial responders in converting a physical stimulus into a developmental signal, offering new insights for improving silkworm breeding efficiency.

## 1. Introduction

Many insects exhibit diapause, a state of developmental arrest that serves as a survival strategy to adapt to natural environmental conditions. This dormancy typically enables insects to endure harsh seasons such as hot summers or cold winters, resuming development and hatching in favorable seasons to access abundant food resources [1,2]. Diapause can occur at various developmental stages, including egg, larval, pupal, and adult phases [3,4].

The silkworm (*Bombyx mori*) is a traditional economically important insect for silk production, with a history of domestication spanning over 5000 years [5,6]. It is a typical example of an egg-diapausing insect, usually entering diapause at the late gastrula stage [7]. The diapause trait in silkworms is also closely associated with climatic differences between northern and southern regions. In colder areas, univoltine and bivoltine varieties, characterized by entering diapause after oviposition, are predominant. In contrast, in warmer southern regions, polyvoltine or facultative varieties have evolved, allowing eggs to bypass diapause and continue developing [8]. In modern sericulture, diapause termination can be artificially induced through methods such as temperature and light control or hydrochloric acid treatment, enabling year-round continuous incubation and rearing [9].

In previous studies, we found that corona treatment similarly effectively terminates diapause in silkworm eggs [10]. Research has indicated that the diapause hormone plays an important role in regulating insect diapause [11]. Although genomic microarrays, RNA-seq, and metabolomic approaches have been applied to compare diapause and non-diapause eggs in an attempt to elucidate the regulatory mechanisms underlying diapause initiation and termination, the molecular networks involved remain poorly understood [9,12,13,14]. In previous research, transcriptomic analysis of early-stage eggs (1–20 h after corona or hydrochloric acid treatment) suggested the potential involvement of pathways such as the FoxO signaling pathway in diapause termination by corona treatment. However, this information remains limited and does not fully reflect the early processes of embryonic diapause termination [15].

To further decipher the molecular mechanisms by which corona treatment terminates diapause, this study employs transcriptomic and proteomic approaches to investigate embryonic development within 0–48 h after corona treatment. The findings are expected to provide valuable insights into identifying key genes and regulatory networks involved in diapause termination, thereby laying a foundation for understanding the molecular mechanisms of embryonic development in silkworm eggs.

## 2. Materials and Methods

### 2.1. Silkworm Materials

The silkworm strain 7532 was bred by the Guangxi Zhuang Autonomous Region Sericulture Technology Promotion Station. Male and female moths were allowed to self-cross for 4 h, after which female moths were placed on silkworm egg paper to lay eggs. The eggs were maintained at 25 °C. Eggs from each parental moth were randomly assigned to control or treatment groups. The control group received no treatment. The samples were collected at 0 h (after laying eggs), 25 h, 36 h, 48 h, 60 h, and 72 h. The corona treatment group (12 kV, 1 min) was treated using GZ-01 (the artificial corona instrument developed by us) after laying eggs for 24 h [10], and samples were collected at 1 h (after corona treatment), 12 h, 24 h, 36 h, and 48 h. Three biological replicates were collected at each time point, each derived from a parental moth. Each sample was divided into two groups and immediately frozen in liquid nitrogen for RNA-seq (about 120 eggs per sample) and proteome (about 120 eggs per sample) analysis.

### 2.2. RNA Extraction, Library Construction, and Sequencing

Total RNA was extracted using a TRIzol reagent kit (Invitrogen, Carlsbad, CA, USA) according to the manufacturer’s protocol. Then, RNA quality was determined using a 5300 Bioanalyzer (Agilent, Santa Clara, CA, USA) and quantified using ND-2000 (NanoDrop Technologies, Wilmington, DE, USA). RNA purification, reverse transcription, library construction, and sequencing were performed at Shanghai Majorbio Bio-pharm Biotechnology Co., Ltd. (Shanghai, China), according to the manufacturer’s instructions. The silkworm egg RNA-seq transcriptome library was prepared following Illumina^®^ Stranded mRNA Prep, Ligation (Illumina, Inc., San Diego, CA, USA). Libraries were size-selected for cDNA target fragments of 300–400 bp using magnetic beads followed by PCR amplification for 10–15 PCR cycles. After being quantified by Qubit 4.0, the sequencing library was performed on the NovaSeq X Plus platform.

### 2.3. RNA-Seq Data Analysis

The raw paired-end reads were trimmed and quality controlled by fastp (version 0.23.4) [16]. The silkworm reference genome and annotation files (version 3.0) were downloaded from https://silkdb.bioinfotoolkits.net/base/download/-1 (accessed on 20 June 2025) [6,17]. Then, clean reads were aligned to the reference genome using HISAT2 (version 2.2.1) with default parameters [18]. The mapped reads of each sample were assembled by StringTie (version 2.2.1) in a reference-based approach for new transcripts [19,20]. The expression level of each transcript was calculated according to the transcripts per million reads (TPM) method. The resulting alignments were used to generate raw read counts for each gene using RSEM [21]. This raw count matrix served as the input for differential expression analysis. Differentially expressed gene (DEG) analysis was conducted using DESeq2 (Version 1.42.0). Significance of differential expression was assessed using the Wald test, and the resulting *p*-values were adjusted for multiple testing via the Benjamini–Hochberg procedure to control the false discovery rate (FDR). Transcripts showing an absolute log_2_ (fold change) value of >1 and an FDR of <0.05 were considered differentially expressed [22].

### 2.4. Proteomic

For proteomic analysis, proteins were extracted and validated by SDS–polyacrylamide gel electrophoresis (SDS-PAGE). The digested peptides were analyzed using liquid chromatography–tandem mass spectrometry (LC-MS/MS) on an Orbitrap Fusion Lumos instrument (Thermo Fisher Scientific, Waltham, MA, USA). Data-independent acquisition (DIA) raw data were acquired on an Orbitrap Astral mass spectrometer. Data processing was performed using Spectronaut (Version 19) with a project-specific spectral library generated from data acquired on an Orbitrap Fusion Lumos. Protein quantification was normalized using median global normalization. Missing values were imputed using a k-nearest neighbors algorithm. Differential expression analysis based on quantitative protein abundance results was performed to identify differentially abundant proteins (DAPs) between the two groups. The statistical test used was an unpaired, two-sided Student’s *t*-test, with the following selection thresholds: absolute log_2_ (fold change) ≥ 0.263 (approximately corresponding to a 20% abundance change) and *p*-value < 0.05.

### 2.5. Function Enrichment Analysis

Trend analysis of the two groups was conducted using Mfuzz (v2.60.0) [23]. Weighted Gene Co-Expression Network Analysis (WGCNA) was conducted using the WGCNA R package (v1.72). Gene expression or protein abundance values were input to construct co-expression networks with the following parameters: a soft-thresholding power of 7, a mergeCutHeight of 0.25, and a min-moduleSize of 30. DEGs/DAPs were further subjected to Gene Ontology functional analysis and Kyoto Encyclopedia of Genes and Genomes (KEGG) pathway analysis at a Bonferroni-corrected *p*-value < 0.05 [24,25,26].

## 3. Results

### 3.1. Corona Treatments Promote the Hatching of Silkworm Eggs

**Figure 1:**
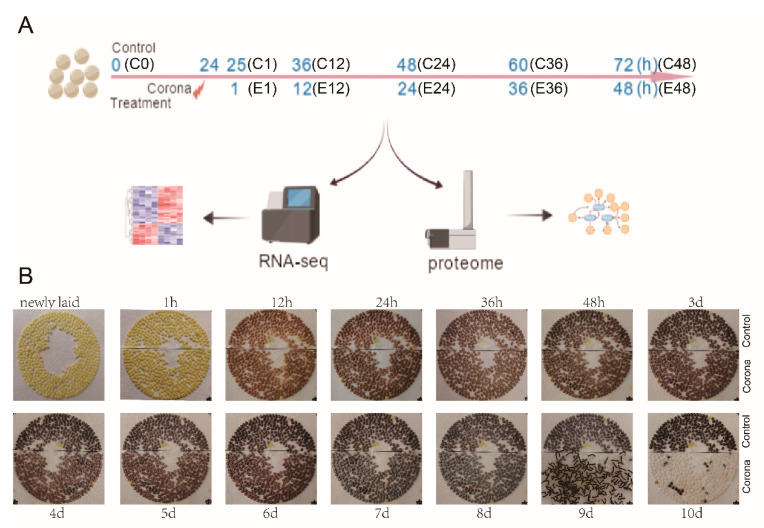
Schematic diagram of the experimental process and comparison of silkworm egg development. (**A**) Schematic diagram of experimental design. (**B**) Images of eggs at different developmental stages between control and corona treatment group. The accelerated hatching phenotype shown was consistently observed across all three independent biological replicates.

To elucidate the molecular mechanisms by which corona treatment accelerates embryonic development in silkworm eggs, newly laid eggs (within 24 h post-oviposition) were subjected to electrically induced corona discharge (12 kV, 1 min). Samples were collected at 1, 12, 24, 36, and 48 h post-treatment (designated E1–E48), with untreated eggs serving as time-matched controls (C0–C48). Each time point included three biological replicates, and all samples were split for parallel transcriptomic and proteomic analyses (Figure 1A).

Morphological assessments revealed marked divergence in embryogenesis between the two groups. By day 9, >80% of corona-treated eggs had completed hatching, whereas none of the control eggs showed signs of hatching (Figure 1B). This observation corroborates previous findings and confirms the efficacy of corona treatment in terminating diapause and accelerating larval hatching [15].

### 3.2. Transcriptional Changes in Silkworm Eggs After Treatment with Corona

First, we constructed 33 transcriptomic libraries. High-throughput sequencing generated approximately 216 GB of raw data, which yielded 215 GB of clean data after quality filtering, averaging 6.5 GB per sample (Supplementary Table S1). These clean reads were then mapped to the silkworm reference genome, resulting in a mapping rate of >94.5% for all samples, with uniquely mapped reads exceeding 90% (Supplementary Table S2). Although >60% of these reads aligned to CDS regions, approximately 4–10% mapped to intergenic and intronic regions, which may include reads from unannotated genomic regions, novel transcripts, or alternative splicing isoforms (Supplementary Table S3). Gene expression levels were quantified using TPM values (Supplementary Table S4).

**Figure 2:**
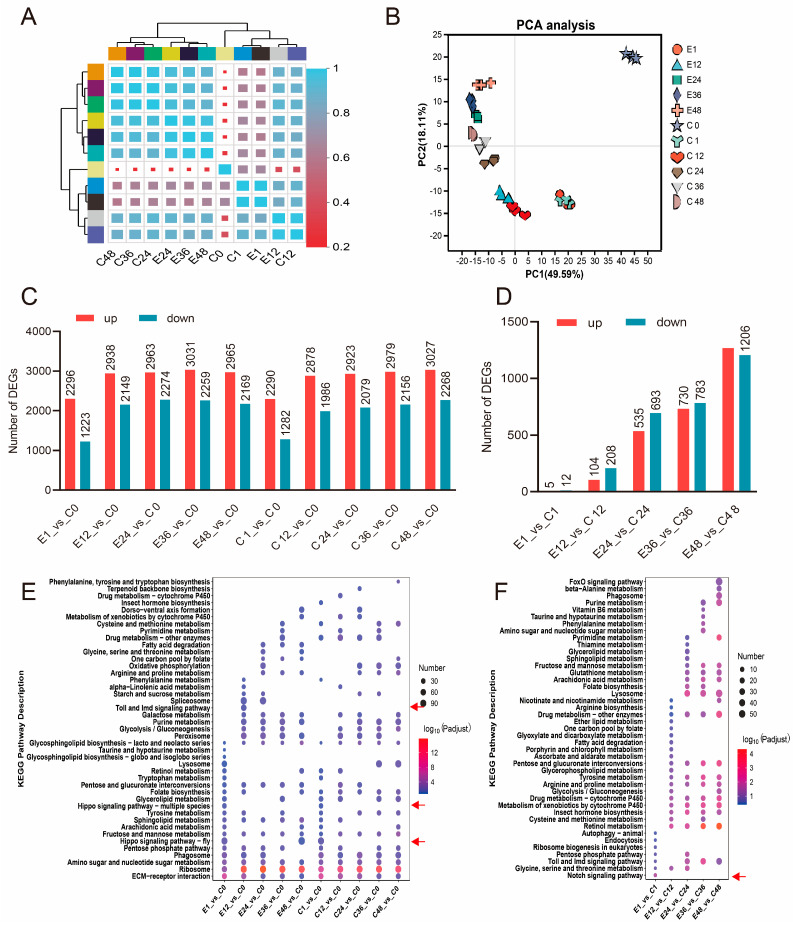
Transcriptome analysis of silkworm eggs after corona treatment. (**A**) Correlation coefficients between gene expression datasets. Red and blue colors indicate correlation coefficients between samples, respectively. (**B**) Principal component analysis (PCA) of transcriptome data from control and electrically treated groups at different time points. Each point represents a sample, and the percentage of variance explained by each principal component is indicated in parentheses. (**C**) Number of DEGs compared to C0 at different time points. The number of upregulated and downregulated genes is shown for each comparison. (**D**) Number of DEGs between control and treatment groups at different time points. (**E**) KEGG enrichment analysis of DEGs in comparison groups in (**C**). Arrows show mainly enrichment in E1-vs-C0, C1-vs-C0, and EC12-vs-C0. (**F**) KEGG enrichment analysis of DEGs in compared groups in (**D**). Arrows show only enrichment in E1-vs-C1. The color and size of the bubbles indicate significant enrichment and gene number, respectively. Pathways were considered significantly enriched at a Bonferroni-adjusted *p*-value < 0.05.

Sample correlation analysis revealed stronger intra-group similarity among 24–48 h samples, whereas 1–12 h samples showed higher inter-group similarity at matched time points. This indicates substantial divergence between 1–12 h and 24–48 h developmental phases across both control and experimental groups, suggesting pronounced divergence in gene expression patterns between control and experimental groups starting at 24 h post-treatment. This potentially indicates altered developmental trajectories in embryos (Figure 2A). Principal component analysis (PCA) corroborated these patterns. Tight clustering of triplicate biological replicates confirmed high reproducibility. Principal components 1 and 2 (PC1 and PC2) accounted for 49.59% and 18.11% of total variance, respectively (Figure 2B).

Differential gene expression analysis relative to C0 revealed comparable magnitudes of transcriptional changes across most time points in both groups. Specifically, while E1_vs_C0 and C1_vs_C0 comparisons yielded approximately 3500 DEGs, all other time points exhibited ~5000 DEGs versus C0. This indicates substantial transcriptomic reprogramming in both control and experimental groups during 24–48 h developmental stages (Figure 2C). Strikingly, pairwise experimental–control comparisons revealed temporally escalating divergence: E1_vs_C1 showed minimal differential expression (5 upregulated; 12 downregulated); E12_vs_C12 contained 104 upregulated and 208 downregulated; E24_vs_C24 exhibited 535 upregulated and 693 downregulated; E36_vs_C36 displayed 730 upregulated and 783 downregulated; and E48_vs_C48 presented 1268 upregulated and 1206 downregulated DEGs. This escalating divergence demonstrates that although both groups undergo extensive transcriptional remodeling post-C0, experimental and control embryos activate fundamentally distinct genetic programs, suggesting divergent developmental trajectories (Figure 2D).

Functional enrichment analysis of DEGs revealed pathway-specific divergence beyond conserved metabolic processes. While core metabolic pathways were enriched across multiple comparisons, significant differences emerged in temporal enrichment patterns. Consistent with correlation and PCA results, transcriptional profiles diverged substantially between experimental and control groups after 24 h. Notably, the 1 h and 12 h timepoints may represent critical regulatory windows for treatment-induced developmental reprogramming. KEGG enrichment demonstrated Hippo signaling pathway enrichment exclusively in E1_vs_C0 and C1_vs_C0 and Toll and Imd signaling pathways uniquely enriched in E12_vs_C0, potentially facilitating enhanced embryonic hatching in treated groups post-12 h (Figure 2E). Pairwise experimental–control comparisons at the same timepoints showed that 12–48 h DEGs were predominantly associated with developmental processes and specialized metabolism, but the Notch signaling pathway enrichment solely in E1_vs_C1, suggesting early developmental modulation (Figure 2F).

### 3.3. Trend of DEGs After Treatment with Corona

**Figure 3:**
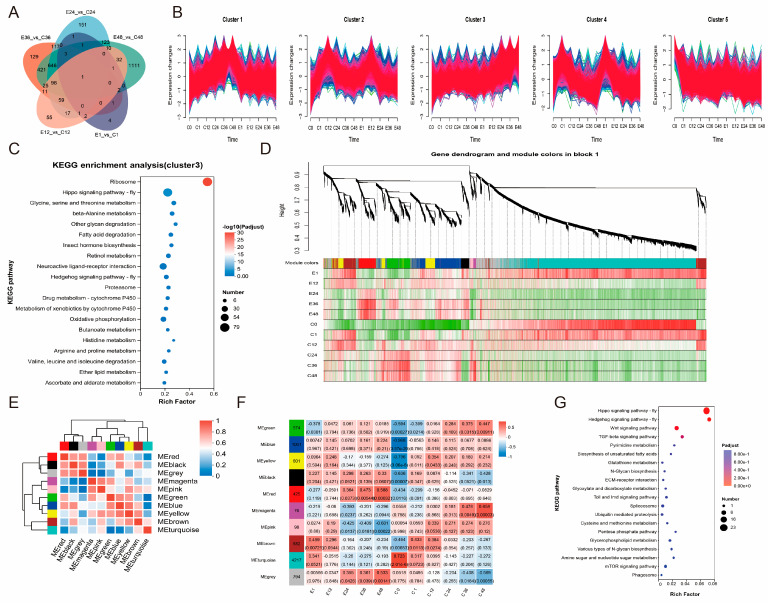
The trend of DEGs in response to corona treatment. (**A**) Venn diagram showing the three comparison groups between control and treatment at five time points. (**B**) Cluster of the DEG response to corona treatment. (**C**) KEGG analysis of cluster 3 genes. The color and size of the bubbles indicate significant enrichment and gene number, respectively. (**D**) Hierarchical cluster tree showing 10 modules of co-expressed genes. Each leaf represents one gene in the tree. Heatmap showing the expression profile in each sample. (**E**) Module correlations. (**F**) Module–trait correlations and corresponding *p*-values. Each row corresponds to a cluster. The left panel shows 10 modules, and the right panel is a color scale for module–trait correlation from –1 to 1. (**G**) KEGG analysis of black module genes. The color and size of the bubbles indicate significant enrichment and gene number, respectively. Pathways were considered significantly enriched at a Bonferroni-adjusted *p*-value < 0.05.

Analysis of DEGs between the experimental and control groups across five time points revealed a progressive increase in the number of time-specific DEGs, while common DEGs were limited, indicating substantial divergence in gene expression patterns among time points (Figure 3A). Trend analysis of gene expression across all samples demonstrated that genes in cluster 3 exhibited minimal variation in the control group but displayed a gradual increase in the experimental group, corresponding with developmental progression (Figure 3B). Functional enrichment analysis of cluster 3 genes revealed significant enrichment in the Hippo signaling pathway, further supporting the potential role of this pathway in promoting embryonic development (Figure 3C). Subsequent WGCNA analysis categorized these genes into 10 modules, with genes in the red module showing progressively upregulated expression (Figure 3D,E). The black module exhibited consistent correlation with the experimental group, whereas it maintained high correlation with the control group only at C1 and C12 (Figure 3F). Enrichment analysis of the black module genes identified significant associations with the Hippo signaling pathway, Hedgehog signaling pathway, and Wnt signaling pathway (Figure 3G).

### 3.4. Protein Content Changes in Silkworm Eggs After Treatment with Corona

**Figure 4:**
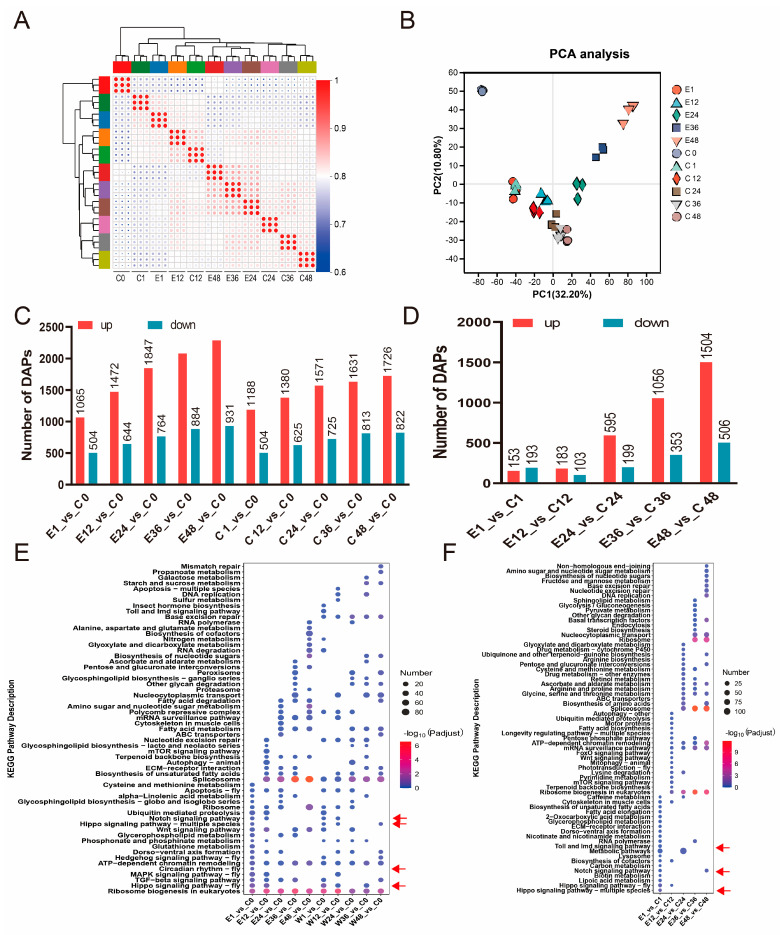
Proteomic analysis of silkworm eggs after corona treatment. (**A**) Correlation coefficients between protein accumulated datasets. Red and blue colors indicate correlation coefficients between samples, respectively. (**B**) Principal component analysis (PCA) of transcriptome data from control and electrically treated groups at different time points. Each point represents a sample, and the percentage of variance explained by each principal component is indicated in parentheses. (**C**) Number of DAPs compared to C0 at different time points. The number of upregulated and downregulated proteins is shown for each comparison. (**D**) Number of DAPs between control and treatment groups at different time points. (**E**) KEGG enrichment analysis of DAPs in comparison groups in (**C**). Arrows show mainly enrichment in E1/C1_vs_C0 and E/C12_vs_C0. (**F**) KEGG enrichment analysis of DAPs in compared groups in (**D**). Arrows show only enrichment in E1_vs_C1. The color and size of the bubbles indicate significant enrichment and gene number, respectively. Pathways were considered significantly enriched at a Bonferroni-adjusted *p*-value < 0.05.

To investigate the effect of corona treatment on protein levels, we further conducted a quantitative proteomic study. A total of 60,697 peptides and 5841 proteins were identified. Analysis of the correlation and reproducibility among protein samples showed results largely consistent with those at the transcriptomic level, with stronger intra-group similarity observed among 24–48 h samples, while 1–12 h samples exhibited higher inter-group similarity at matched time points (Figure 4A,B). However, unlike the transcriptomic results where the number of DEGs compared to C0 remained relatively stable in the 24–48 h stages, the number of DAPs between samples at different time points and C0 showed a gradually increasing trend in both the treatment and control groups (Figure 4C, Supplementary Table S5). Pairwise comparisons between the treatment and control groups at each time point revealed that the number of DAPs remained relatively low (approximately 300) at 1–12 h (E1_vs_C1, E12_vs_C12) but increased significantly to 794–2010 at 24–48 h (Figure 4D), which may be associated with the activation of more embryonic-development-related genes in the 24–48 h stages.

KEGG enrichment analysis of DAPs from each comparison group in Figure 4C showed that pathways significantly enriched at the 1–12 h stages (C/E1_vs_C0, C/E12_vs_C0) included the Hippo signaling pathway, circadian rhythm, Notch signaling pathway, and mTOR signaling pathway (Figure 4E). Meanwhile, enrichment analysis of DAPs from the comparison groups in Figure 4D indicated that pathways such as the Hippo signaling pathway, Notch signaling pathway, and Toll and Imd signaling pathways were significantly enriched at 1 h post-treatment, while the mTOR signaling pathway, FoxO signaling pathway, and Wnt signaling pathway were prominently enriched at 12 h post-treatment (Figure 4F).

### 3.5. Functional Enrichment of DAPs After Treatment with Corona

**Figure 5:**
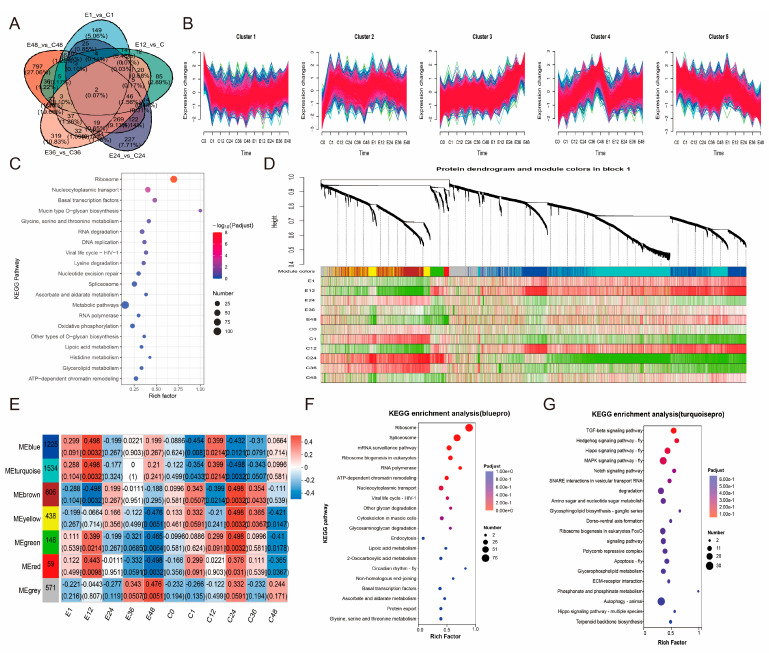
The trend of DAPs in response to corona treatment. (**A**) Venn diagram showing the three comparison groups between control and treatment at five time points. (**B**) Cluster of the DAP response corona treatment. (**C**) KEGG analysis of cluster 3 proteins. The color and size of the bubbles indicate significant enrichment and gene number, respectively. (**D**) Hierarchical cluster tree showing 7 modules of co-expressed genes. Each leaf represents one gene in the tree. Heatmap showing the expression profile in each sample. (**E**) Module correlations. (**F**) KEGG analysis of blue module proteins. (**G**) KEGG analysis of turquoise module proteins. The color and size of the bubbles indicate significant enrichment and gene number, respectively. Pathways were considered significantly enriched at a Bonferroni-adjusted *p*-value < 0.05.

Similarly, analysis of time-point-specific DAPs across comparison groups revealed that their numbers were relatively low at 1 h and 12 h (149 and 85, respectively) and progressively increased from 227 to 797 between 24 h and 48 h (Figure 5A), a trend consistent with the earlier observed changes in the number of DEGs. Analysis of abundance patterns of these proteins showed that those in cluster 3 exhibited limited variation across time points in the control group but increased steadily over time in the treatment group (Figure 5B). KEGG enrichment analysis of cluster 3 proteins indicated significant enrichment in pathways such as Ribosome, Nucleocytoplasmic transport, and Basal transcription factors (Figure 5C). Based on WGCNA, these proteins were categorized into seven major modules (Figure 5D). Among these, the grey module showed a gradually increasing correlation with longer treatment duration, while the turquoise and blue modules were highly correlated with the 1–12 h treatment time points (1 h and 12 h), suggesting their involvement in initial response mechanisms (Figure 5E). KEGG enrichment analysis further revealed that the blue module was primarily associated with fundamental biological processes, whereas the turquoise module was significantly enriched in pathways such as the TGF-beta signaling pathway, Hedgehog signaling pathway, and Hippo signaling pathway (Figure 5F,G), showing considerable consistency with transcriptomic findings.

### 3.6. Correlations of Transcriptomic and Proteomic Data

**Figure 6:**
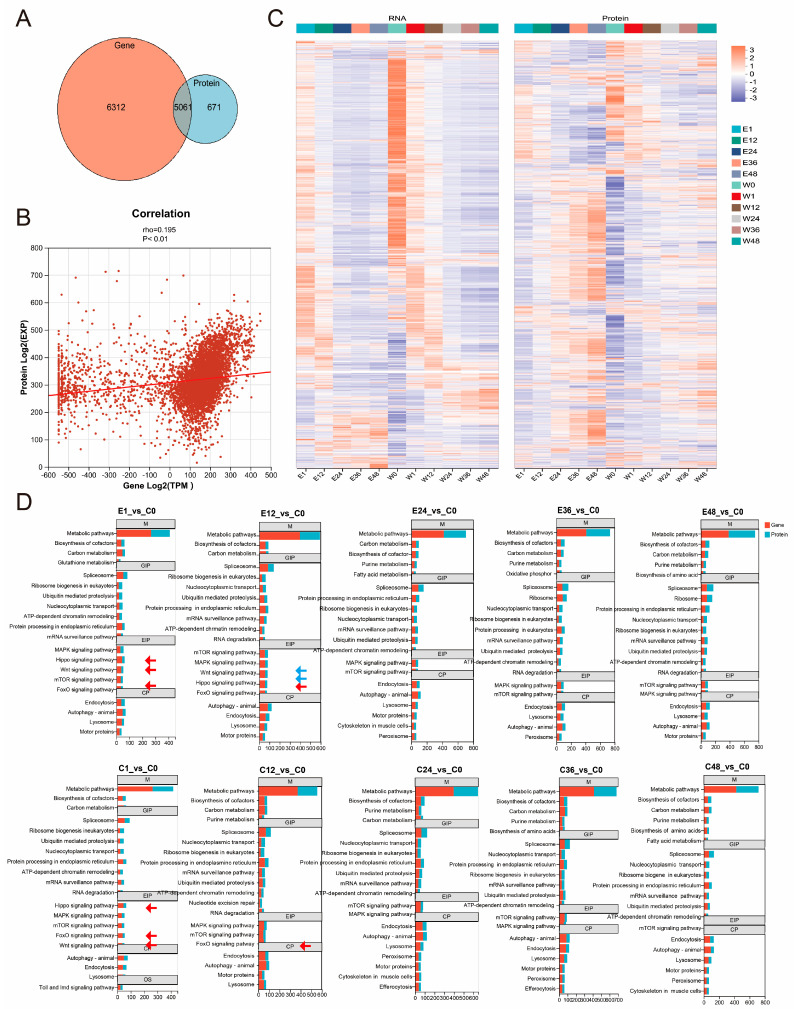
Correlations analysis of transcriptomic and proteomic data. (**A**) Venn diagram showing the data between transcriptomic and proteomic. (**B**) Spearman’s correlation coefficient analysis of transcriptomic and proteomic data. (**C**) Heatmap of gene expression profile and protein abundance. (**D**) DEGs/DAPs significant enrichment trend in multiple compared groups. M, Metabolism; GIP, Genetic Information Processing; EIP, Environmental Information Processing; CP, Cellular Processes; OS, Organismal Systems. Red box means gene enrichment results, blue box shows protein enrichment results. Red arrows showed Hippo, Wnt, and FoxO signaling pathways, blue arrows showed Hippo and Wnt signaling pathways only significant enrichment in E12_vs_C0.

Integration of the two omics datasets revealed 5061 common protein-coding genes, along with 6312 genes unique to the transcriptome and 671 proteins unique to the proteome (Figure 6A). These results indicate that approximately half of the expressed genes detected at the transcript level had corresponding protein products identified, while a substantial number of transcribed genes still lack protein evidence. The relatively low correlation between the two datasets, with a Spearman correlation coefficient (rho) of 0.195 (*p* < 0.01), further supports a modest concordance between transcript and protein abundances (Figure 6B). This discrepancy may be attributed to post-transcriptional and post-translational regulatory mechanisms. Consistently, heatmap analysis revealed considerable differences in expression patterns between the transcriptomic and proteomic profiles (Figure 6C).

Based on previous findings, we preliminarily concluded that corona treatment primarily acts at the 1–12 h stages to terminate diapause and promote embryonic development. Further integrated analysis of multi-group gene and protein expression data within the category of Environmental Information Processing indicated that, among the five pathways significantly enriched at 1–12 h time points, only the Hippo, Wnt, and FoxO signaling pathways were prominently enriched as early as 1 h post-treatment (including E1_vs_C0 and C1_vs_C0, indicated by red arrows). By contrast, at 12 h post-treatment, the Hippo and Wnt signaling pathways remained significantly enriched only in the treated group (E12_vs_C0, blue arrow) but not in the control group (C12_vs_C0). This suggests that the Hippo and Wnt signaling pathways may play critical roles in promoting continued embryonic development in silkworms following corona treatment (Figure 6D). Importantly, the sustained enrichment of these pathways, specifically in the E12_vs_C0 comparison, but not in C12_vs_C0, may provide evidence that their activation is a consequence of the corona treatment.

### 3.7. Hippo and Wnt Signaling Pathways Involved in Embryonic Development 

**Figure 7:**
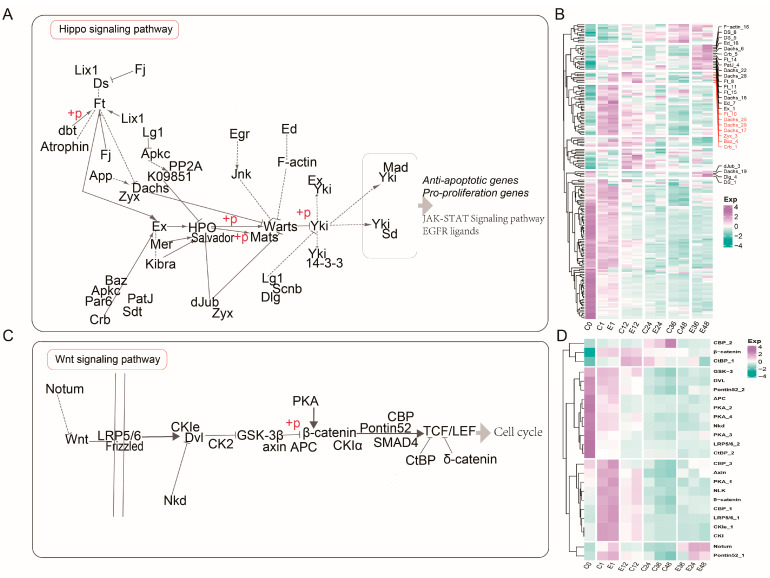
DEGs DAPs in Hippo and Wnt signaling pathways. (**A**) Hippo and Wnt signaling pathways. (**B**) The heatmap of DEGs in Hippo signaling pathways. (**C**) Hippo and Wnt signaling pathways. (**D**) The heatmap of DEGs in Wnt signaling pathways. Log_2_-scaled TPMs in different time points are presented. Low to high expression is indicated by a change in color from green to purple.

As previously indicated, the Hippo and Wnt signaling pathways were the most prominently differentially enriched pathways between the treatment and control groups in the 1–12 h stages. Analysis of key enriched genes in the Hippo signaling pathway revealed that samples from the same time points clustered closely together during the early phase of treatment (0–24 h), such as C1 with E1, C12 with E12, and C24 with E24. However, gene expression diverged between the treatment and control groups at 36–48 h, with control samples (C36 and C48) and treated samples (E36 and E48) forming distinct clusters. Notably, two gene clusters showed markedly elevated expression in the E36 and E48 treatment groups, including genes such as F-actin16, DS_5/6, Ed_7/16, Dachs_6/16/22/28, and Ft8/11/14/15. In addition, another cluster comprising Dachs_17/25/29, Ft10, Zyx_3, Baz_4, and Crb_1 showed substantially higher expression in the treatment group as early as 1 h post-treatment, suggesting their potential role in the early regulation of the Hippo signaling pathway (Figure 7A,B and Supplementary Table S6). This pathway is primarily involved in regulating anti-apoptotic and pro-proliferation genes, with downstream activities including the JAK-STAT signaling pathway.

The Wnt signaling pathway is closely associated with cell cycle regulation; during 0–12 h, treated and control samples at the same time points clustered together. However, unlike the Hippo pathway, separation between treatment and control groups became apparent starting at 24 h, and the three formed distinct clusters at 24–48 h accordingly. Notably, expression of Notum and Pontin52 was significantly higher in the treatment group at the early time point (1 h), and this elevated expression persisted in the 24–48 h treatment samples. This implies that Notum and Pontin52 may act as early regulatory genes in the Wnt signaling pathway and that this pathway may respond to corona treatment earlier than the Hippo pathway, participating in the regulation of silkworm embryonic development (Figure 7C,D and Supplementary Table S6).

## 4. Discussion

In this study, leveraging high-temporal-resolution transcriptomic and proteomic analyses delineates the early molecular events underpinning corona-discharge-induced diapause termination. In contrast to previous findings, which highlighted the activation of the FoxO signaling pathway at 6 h post-treatment [15], the present data reveal that the Hippo and Wnt signaling pathways are specifically activated within 1 h of treatment. This discovery significantly advances the initial response window and positions these pathways as upstream master regulators potentially responsible for sensing the corona stimulus and initiating the developmental program. The concordance between multi-omics datasets and the reinforcing results from WGCNA module analysis substantiate that the early activation of the Hippo and Wnt pathways is a central, coordinated event. These insights provide a novel perspective on the initiation of diapause termination, shifting the focus to these immediate–early signaling cascades.

The Hippo signaling pathway exhibits a high degree of evolutionary conservation in both insect and mammalian systems and is recognized as a central regulator of cell proliferation, apoptosis, and organ size, and it is a compelling candidate for driving the exit from diapause [27,28,29,30]. We identified two temporally distinct gene expression clusters within this pathway. One cluster, including genes such as Dachs_17/25/29 and Ft_10, was significantly upregulated as early as 1 h post-treatment. As upstream components, these genes may serve as primary sensors for corona-induced cues, such as cytoskeletal rearrangement or mechanical stress. Subsequently, second clusters containing genes like F-actin_16 and Ed_7/16 exhibited sustained high expression at 36–48 h, likely driving the pathway’s downstream effects on anti-apoptotic and pro-proliferation genes to facilitate a stable exit from the cell cycle arrest characteristic of diapause [7]. Furthermore, the pathway’s downstream connection to JAK-STAT signaling integrates it into a broader developmental regulatory network.

The enrichment of the Hippo signaling pathway in both E1_vs_C0 and C1_vs_C0 comparisons suggests that certain developmental machinery remains in a poised state during diapause. This indicates that comparisons against the oviposition baseline alone cannot distinguish treatment-specific effects from general developmental progression. Therefore, our inference also relies on time-matched contrasts (E vs. C) and the subsequent trajectory divergence. Critically, while early direct comparisons showed minimal differences, the Hippo and Wnt pathways exhibited sustained and exclusive enrichment in the treated group at 12 h (E12_vs_C0, Figure 6D) and a complete separation of gene expression clusters by 36–48 h (Figure 7). This demonstrates that corona treatment does not merely accelerate a latent program but orchestrates a sustained and divergent reactivation of these pathways, ultimately driving diapause termination.

Functioning in parallel, the Wnt signaling pathway is a cornerstone of embryonic development and cell cycle regulation, which may emerge as another critical early responder [31]. In multicellular organisms, Wnt proteins govern stem and progenitor cell renewal and differentiation to regulate embryonic development, adult tissue homeostasis, and tissue regeneration [32,33]. Notably, key pathway components such as Notum (a Wnt signaling modulator) and Pontin52 (involved in transcriptional regulation) were strongly upregulated in the treatment group at the 1 h mark, with elevated expression persisting thereafter. This suggests that these genes may act as pioneer factors initiating developmental reprogramming. Interestingly, the Wnt pathway demonstrated a faster divergence between treated and control groups, becoming apparent at 24 h compared to the later divergence of the Hippo pathway. This indicates that Wnt may be more directly involved in initiating cell cycle progression. We thus propose a testable synergistic model in which the corona stimulus may rapidly “unlock” the cell cycle via the Wnt signaling pathway, while concurrently, the Hippo pathway could establish a cellular environment conducive to proliferation and antagonistic to apoptosis, together potentially orchestrating the resumption of development.

The integrated multi-omics approach revealed a moderate global correlation between transcriptomic and proteomic profiles (Spearman’s ρ = 0.195). This is consistent with the complex, multi-layered regulation governing rapid developmental transitions, where protein abundance is determined by factors beyond mRNA levels, including translational efficiency and protein turnover [34,35].

Our integrated multi-omics approach revealed a moderate correlation between transcriptomic and proteomic profiles, underscoring the complexity of post-transcriptional regulation during this dynamic process. An important finding requiring explanation is the significant FoxO pathway enrichment at early time points both in control and treatment groups in this study, which contrasts with previous report. We posit this is not a contradiction but a consequence of the enhanced temporal resolution of the current experimental design. The previous study had a sampling gap between 1 and 6 h, potentially missing the earliest Hippo/Wnt responses. Therefore, we propose a hierarchical response model: the corona stimulus first triggers an immediate–early response (e.g., Hippo, Wnt), which then guides a mid-term regulatory phase (e.g., FoxO), ultimately converging on the effector execution stage of metabolic restructuring [36].

While this study delineates the early signaling response, we acknowledge that the proteomic analysis was conducted without multiple-testing correction to maximize sensitivity, which may increase the false-positive risk, particularly at early time points. Therefore, the functional necessity of the identified pioneer genes (Notum, Pontin52, Dachs) awaits direct validation through targeted genetic approaches such as RNA interference and CRISPR/Cas9-mediated knockout.

## 5. Conclusions

In summary, our integrated multi-omics analysis suggests that the Hippo and Wnt signaling pathways act as early initiators of corona-induced diapause termination in silkworm, preceding the FoxO response. We propose a working model wherein their concurrent activation may reprogram the cell cycle to restart development. These findings provide a correlative framework and pinpoint key candidate genes (e.g., *Notum*, *Pontin52*, *Dachs*) for functional validation. Future research should employ genetic approaches in silkworm embryos to test their necessity, thereby establishing causality and fully elucidating the underlying mechanism. These investigations will solidify the molecular framework for understanding how physical stimuli terminate insect diapause.

## Data Availability

RNA-seq raw data are available at NGDC (https://ngdc.cncb.ac.cn/) BioProject (accession number PRJCA050010), and proteome raw data are available at iProX (https://www.iprox.cn) (accession number IPX0014140001).

## Supplementary Materials

The following supporting information can be downloaded at: https://www.mdpi.com/article/10.3390/insects17010123/s1, Table S1. Statistics of RNA-seq information. Table S2. The statistics of mapping the reference genome. Table S3. The mapping region statistics. Table S4. The TPM value of all genes. Table S5. The protein abundance value. Table S6. The gene enrichment in Hippo and Wnt signaling pathways.

## Abbreviations

The following abbreviations are used in this manuscript:

TPM	Transcripts per million reads
DEGs	Differentially expressed genes
PAGE	Polyacrylamide gel electrophoresis
LC-MS/MS	Liquid chromatography–tandem mass spectrometry
DIA	Data-independent acquisition
DAPs	Differentially abundant proteins
FDR	False discovery rate
WGCNA	Weighted Gene Co-Expression Network Analysis
KEGG	Kyoto Encyclopedia of Genes and Genomes
PCA	Principal component analysis
